# The Mercurial Bath

**Published:** 1855-08

**Authors:** 


### THE MERCURIAL VAPOR BATH.

Mention having been made in another part of the Journal of the Mercurial Vapor Bath, we have thought it might prove acceptable to our readers to give the following extract from Mr. Parker's work on Syphilis relating to the subject. It is our purpose to prepare for an early number of the Journal an account of the cases that we have submitted to this mode of treatment.

D. W. Y.

The patient is placed on a chair, and covered with an oil-cloth, lined with flannel, which is supported by a proper frame-work. Under the chair are placed a copper bath, containing water, and a metal plate, on which is put from one to three drachms of the bisulphuret of mercury, or the same quantity of the gray oxide, or the binoxide. Under each of these a spirit-lamp. The patient is thus exposed to the influence of three agents, heated air, common steam, and the vapor of mercury, which is thus applied to the whole surface of the body in a moist state. After the patient has remained in the bath from five to ten minutes perspiration generally commences, and by the end of twenty or thirty minutes, beyond which I do not prolong the bath, it is generally excessive. The lamps are now removed, and the temperature gradually allowed to sink; when the patient has become moderately cool, the coverings are removed, and the body rubbed dry; the patient is suffered to repose in an arm-chair for a short time, during which he drinks a cup of warm decoction of guaiacum or sarsaparilla.

The apparatus requires some modification and management to particular cases. Where it is wanted to induce a quick and decided action, the whole power of the bath should be brought into operation, and the largest quantity of mercury should be emplyed. In rapidly-spreading ulcers this is required. Again, in chronic skin or throat diseases, where a powerful action would rather oppress the patient than cure his disease, the power of the bath should be modified, and not so great a heat or so much mercury employed. This is accomplished by using smaller spirit-lamps, or, when perspiration has once been induced, by the removal of one lamp, leaving the patient thus exposed for a time to the mercurial vapor alone. This should be done where the patient has been broken down by long-continued disease, in bad or weak subjects, or where a more prolonged action is required to eradicate the more deep-seated effects of the venereal poison, as in diseases of the bones, or indurations on the penis. Each particular case would require a greater or less modification of this kind. The form of mercurial employed is also of consequence. In skin diseases the bisulphuret is to be preferred, in diseases of the throat or nose the gray oxide, or binoxide is better, because the patient can bear the head immersed without sneezing or coughing, which he cannot do when the bisulphuret is used.

I am in the habit of using four mercurial preparations for the baths; the bisulphuret of mercury, the binoxide of mercury, the gray or black oxide, and the iodide. These may be used singly or combined in different ways, to suit the peculiarities or emergencies of each particular case. The first three preparations are milder than the last, and from half a drachm to four drachms may be used for a bath with perfect safety. In one case half an ounce was used for each bath, and two applications were sufficient to bring the system fully under the influence of the remedy. The iodide must be used in smaller quantities: nearly the whole of this preparation is rapidly converted into vapor, and, unlike all the other preparations, leaves scarcely any ash behind it. From five grains to half a drachm of the iodide is sufficient, and it is better to use it in small quantities, mixed with a larger quantity of either of the other preparations. In affections of the testes (sarcocele) and of the bones (the various forms of ostitis, or periostitis) a combination of a scruple of the iodide, and one or two drachms of the bisulphuret or binoxide would be a proper form. For local application to the cavities of the nose or mouth, a few grains only should be employed, as the vapor of the iodide of mercury is more irritating and more powerful than that of either of the other preparations I have mentioned.

A short preparatory treatment should be adopted before using the baths. The bowels should be kept free, and the use of wine, spirits, etc., prohibited. The patient should be free from fever, the tongue clean, and the freedom from organic diseases, such as those of the heart and lungs, more particularly, should be ascertained. Should such, or other complications be present, they might require modifications of treatment, but would not prevent its employ, as this is not only the most certain, but the safest way of curing all forms of constitutional syphilis.

This plan of treatment does not commonly require that the patient should forego his ordinary occupations of business, or that he should be confined to the house during its use. It must be admitted that its effects would be accelerated by confinement to bed, or to a couch in a moderately-warm room; but this is by no means imperative, and I have very rarely advised it, except in such cases where exposure or exercise would be positively mischievous, as in cases of sloughing, or rapidly-spreading ulcers in the throat or elsewhere.

The diet should be light, nutritious, and unstimulating; milk, chocolate or cocoa, night and morning; animal food for dinner, with weak wine and water. Where the patient has been reduced by mercury given internally, or by a combination of syphilis and mercury, the diet may be more nutritious; but stimulants should be avoided. Smoking must be prohibited, particularly in disease of the throat and nose.

In a great majority of cases the moist mercurial vapor, employed as I have directed, is capable of curing the disease without the assistance of internal medicine; but the cure is generally expedited and rendered more certain by the administration of the latter in small quantities. The treatment is always assisted by the decoction of sarsaparilla or guaiacum, drank warm night and morning, and immediately after leaving the bath. I prefer the latter, the compound decoction, made according to the formula of the Edinburgh Pharmacopœia. Where other medicines are required to assist the treatment, and I allude particularly to the various preparations of mercury, it is surprising how small a quantity is required when the patient is using the vapor. I have known several instances where diseases which have been rebellious to large quantities of mercury, given for long periods, yield immediately the baths were employed. The effects of mercury upon the system become very quickly manifest under the influence of the baths, when the system had previously resisted this influence. When I employ mercury internally, during the use of the baths, it is either under the form of the biniodide, or bichloride given in solution in small quanties, not exceeding the twentieth of a grain for a dose. The use of this medicine in drachm doses of the ointment in form of friction, in five grains of blue-pill or calomel, two or three times a day, under the old plan of treating venereal diseases by mercury, can never be required, except it is wished to break up the health and constitution of the patient. How many have never recovered from internal mercurial treatments of the kind. I never saw the most delicate patient, either male or female, whose health was injured for one hour under the plan I recommend, and I have very rarely seen a disease that has not been cured. The experience derived from the treatment of many thousand cases warrants me in speaking thus positively on the subject.

The time occupied in the cure of venereal diseases by the mercurial vapor-bath is vastly less than that consumed by any other kind of treatment: its effects are commonly immediate, one full bath very frequently making at once an impression on the disease. Where the hair has been falling rapidly, one bath has arrested this: ulcers which have been rapidly spreading have been rendered stationary by one bath. After two or three baths, the improvement is in most instances marked; and the cure is effected in one-fourth, or even one-sixth of the time required for the success of ordinary treatments. The nature of the cases determines the time occupied in the cure. In superficial skin diseases, or superficial ulcers of the nose and throat, the cure is very rapid. I have constantly known affections of this kind entirely cured in a fortnight or three weeks, with pleasure rather than inconvenience to the patients. In enlargements of the bones and testes, indurations of the penis, persistent induration of the cicatrix of a primary sore, the cure is necessarily more tedious; the change of structure produced in such diseases must have time for removal: nevertheless, in these cases, which require months of treatment, under common circumstances, and which are not unfrequently considered or given up as incurable, the moist mercurial vapor will do more in a month than any other treatment in six. I have known cases of induration of the penis removed in three or four weeks, which have not shown the slightest disposition to amendment after months of ordinary internal treatment.

All authors and all surgeons conversant with the treatment of syphilitic diseases, admit the frequency of relapses under ordinary treatments; hence constitutional diseases are to be feared as the result of primary ulcers; and when one form of constitutional taint has been apparently cured, it is commonly, after a time, succeeded by another. Thus venereal diseases run through those phases or grades which have been termed primary, secondary, or tertiary; and it has been a primary object in all plans of treatment to have recourse to those which, while they cure the disease then present in the best manner, and with the least risk, shall prevent the occurrence of future diseases under another form. Relapses will occasionally occur under all forms of treatment, but I will undertake positively to state, that they are by far less frequent and important under this plan of treatment than any other; and when they do occur, are trivial, and yield with great certainty to a second application of the vapor.

The effects of the mercurial vapor-bath upon the patient vary under different circumstances. If the general health of the patient be apparently good, and we have to control a single isolated symptom of disease, such as a primary sore, an enlarged testis, or an indurated cicatrix, and the baths be used too frequently, the patient would become a little languid, and probably a little thinner; this would be avoided by properly timing the intervals between the baths. Should the patient be laboring under general constitutional taint, and exhibit as local symptoms loss of hair, sore throat, ulcers of the nose, or skin diseases, he almost invariably gets fat under the treatment. The mouth is commonly affected, after using four or six baths, more quickly if the head be immersed, which is better: some patient can bear the head in the bath for five, ten, or even twenty minutes without inconvenience: patients vary in this particular; and it depends very much on the form of mercurial employed. The gums, when affected, are red, elevated, and tender, but the baths never produce salivation, or ulceration of the mouth.

Some forms of constitutional sy[illegible]ore readily yield to the use of the vapor than oth[illegible]ome are cured with an extraordinary degree of rapidity, and are perfectly cured, which is proved by their not having relapsed, or presented a fresh venereal symptom after many years. These forms are superficial diseases of the skin, loss of hair, superficial ulcerations of the nose and throat.

Some varieties require a longer treatment, as diseases of the deeper-seated parts of the skin, some forms of ulceration, diseases of the testicles and of the bones.

To all forms of constitutional syphilitic disease the treatment by vapor is applicable, and beyond all doubt the most speedy, certain, and safe remedy that can be employed; yet there are some forms of disease which yield with greater rapidity than others. That which gives way with the greatest difficulty is the induration which succeeds to the healing of a primary sore. I do not mean that soft fullness which is sometimes found in such situations, but that cartilaginous hardness which is met with under the skin, and which is sure, sooner or later, to end in local or constitutional mischief. I have seen cases which have resisted all modes of treatment but the baths; to these they yield but slowly, but they do yield, and with certainty, after other plans of treatment have been followed for months without success, or with but partial amendment.

One or two objections have been raised to this plan of treatment by the reviewers of the last edition of this work. These are easily answered, and would never have been made had those gentlemen been familiar with its practical working. The chief objection which has been raised, is that it is unmanageable, and the quantity of mercury introduced into the system cannot be regulated, and that rapid and severe salivation might occur. For nearly twenty years I have administered, or superintended the administration of this bath, from four to six times every day, and I have never seen one case where such an effect has been produced.

The analogy has been made with the dry fume, which sometimes has produced such an effect: the mixture and dilution of the vapors of mercury with common steam, and the sweating induced by the bath, entirely removes any fear of this kind, and I would stake my reputation that with proper management it cannot occur.

I must not be understood to say that I consider or recommend the mercurial vapor bath as a specific remedy in all forms of constitutional syphilis, but I repeat that it is the most powerful therapeutic agent in the removal of disease, and the least harmful to the constitution of the patient of any remedy with which I am acquainted; neither am I so prejudiced in favor of this remedy as to reject the assistance of all others, which, as we shall presently see, when associated with it, under certain circumstances, produce the best effects, but which effects, I am bound to say, would not, under many circumstances, occur without the assistance of the vapor, since in numerous instances these remedies have failed in curing the disease when used alone. The profuse sweating induced by the bath, prevents the accumulation of either iodine or mercury in the system, and thus contributes materially to the preservation of the constitution of the patient.

